# One-Step Hydrothermal Synthesis of Cu_2_ZnSnS_4_ Nanoparticles as an Efficient Visible Light Photocatalyst for the Degradation of Congo Red Azo Dye

**DOI:** 10.3390/nano13111731

**Published:** 2023-05-25

**Authors:** Rodrigo Henríquez, Paula Salazar Nogales, Paula Grez Moreno, Eduardo Muñoz Cartagena, Patricio Leyton Bongiorno, Elena Navarrete-Astorga, Enrique A. Dalchiele

**Affiliations:** 1 Instituto de Química, Facultad de Ciencias, Pontificia Universidad Católica de Valparaíso, Casilla 4059, Valparaíso 2340000, Chile; paula.salazar@pucv.cl (P.S.N.); paula.grez@pucv.cl (P.G.M.); eduardo.munoz.c@pucv.cl (E.M.C.); patricio.leyton@pucv.cl (P.L.B.); 2 Laboratorio de Materiales y Superficie, Departamento de Física Aplicada I, Universidad de Málaga, 29071 Málaga, Spain; enavarrete@uma.es; 3 Instituto de Física, Facultad de Ingeniería, Herrera y Reissig 565, C.C. 30, Montevideo 11000, Uruguay; dalchiel@fing.edu.uy

**Keywords:** CZTS, hydrothermal, Congo red azo dye, photocatalysis

## Abstract

A hydrothermal method was successfully employed to synthesize kesterite Cu_2_ZnSnS_4_ (CZTS) nanoparticles. X-ray diffraction (XRD), Raman spectroscopy, X-ray photoelectron spectroscopy (XPS), field-emission scanning electron microscopy (FE-SEM), energy-dispersive X-ray spectroscopy (EDS), transmission electron microscopy (TEM), and optical ultraviolet-visible (UV-vis) spectroscopy were used for characterization of structural, chemical, morphological, and optical properties. XRD results confirmed that a nanocrystalline CZTS phase corresponding to the kesterite structure was formed. Raman analysis confirmed the existence of single pure phase CZTS. XPS results revealed the oxidation states as Cu^+^, Zn^2+^, Sn^4+^, and S^2−^. FESEM and TEM micrograph images revealed the presence of nanoparticles with average sizes between 7 nm to 60 nm. The synthesized CZTS nanoparticles bandgap was found to be 1.5 eV which is optimal for solar photocatalytic degradation applications. The properties as a semiconductor material were evaluated through the Mott–Schottky analysis. The photocatalytic activity of CZTS has been investigated through photodegradation of Congo red azo dye solution under solar simulation light irradiation, proving to be an excellent photo-catalyst for CR where 90.2% degradation could be achieved in just 60 min. Furthermore, the prepared CZTS was reusable and can be repeatedly used to remove Congo red dye from aqueous solutions.

## 1. Introduction

In the search of green semiconducting photovoltaic absorber materials alternative to the conventional ones (i.e., CdTe and Cu(In,Ga)Se_2_), in recent years the quaternary copper compound having the kesterite structure Cu_2_ZnSnS_4_ (CZTS) has emerged as a potential candidate to replace them for next generation solar cells [1,2,3,4,5,6,7]. In addition to its optical and electronic properties, such as an optimal direct band gap (between 1.4 and 1.6 eV), a high absorption coefficient (~10^4^ cm^−1^) (which allows for effective absorption of the incident photons in absorbers with thicknesses of a few microns), an intrinsic *p*-type conductivity, a thermodynamically stable structure, and a three-dimensional symmetry of carrier transport, it is mainly composed of earth-abundant and nontoxic elements (such as Cu, Zn, and Sn) [1,2,3,4,5]. Through theoretical simulations and device modeling, it was concluded that after optimization of a typical solar cell device, CZTS showed excellent solar-to-electrical conversion performance, with an efficiency of 28.2%, compared to CdTe (20.41%) and Cu(In,Ga)Se_2_ (21.41%) [8]. CZTS solar cells have reached 12.6% efficiency, which is still far below the theoretical limit, indicating the efficiency potential of kesterite has not been fully exploited [1,2].

Asides from the early and conventional application of CZTS compound in thin film solar cells [1,2,3,4,5,9], more recently, other ones outside its conventional usage, such as in charge-transfer layers, sensors, thermoelectric devices, and water splitting, have now been reported in the literature [10]. For instance, Yokoyama et al. first demonstrated in 2010 the application of CZTS as a photocathode [10,11]. From this work, CZTS became extensively employed as a photocathode for the production of hydrogen from solar-assisted water splitting [10]. Moreover, it has been proven that CZTS films exhibit excellent photoreactivity and photocatalytic performance [12,13,14]. In recent years, as a promising solar-driven photocatalyst, the CZTS compound has been recognized and intensively studied due to its excellent performances in air purification, and water remediation of organic pollutants and industrial wastes [12,15,16,17,18,19,20,21,22].

As it is well known, most of the properties of nanomaterials are very much dependent on their size, shape, and dimensionality. Further, it is possible to tune their physical and optical properties by varying the nanocrystal size and various synthesis parameters [13,17]. In this sense, higher photocatalytic activity has been exhibited by CZTS in its nano form rather than in its bulk counterpart [17]. Moreover, it has been reported that CZTS with different morphologies has different adsorption and degradation rates of organic pollutants [23].

To produce CZTSs with respectable photocatalytic and photoelectrochemical capabilities, several techniques have been established to date [5]. Several vacuum and non-vacuum thin film deposition techniques, as well as the direct production of kesterite nanocrystals, have been extensively researched among these approaches [5]. In fact, several fabrication techniques have been developed as effective CZTS nanocrystal synthesis methods, such as hot injection, hydrothermal/solvothermal, and microwave-assisted chemical synthesis [5]. Among various wet-chemical processes, hydrothermal/solvothermal CZTS nanocrystal synthesis is a widely favored approach for the controlled production of kesterite nanocrystals [5,24,25]. Moreover, this process is not limited by the temperature and pressure of the vessel, and the reaction does not require the nitrogen atmosphere and refluxing, which on the other hand are highly important parameters for the hot-injection synthetic route [26].

On the other hand, Congo red (CR) is a synthetic azo-anionic dye that has applications in the textile industry and tests for medical diagnosis. However, CR is a known human carcinogen that can also bring various harmful effects to human health [27,28,29,30]. Due to this, several works have been developed to study its elimination from aqueous effluents [31,32,33].

In the present work, Cu_2_ZnSnS_4_ nanoparticles have been synthesized by the hydrothermal technique. Further, structural, morphological, surface chemical study, optical and optoelectronic characterization of these nanostructures has been carried out. The synthesized sample has been used as a photocatalyst to degrade Congo red azo dye to evaluate the photocatalytic performance of CZTS. Moreover, to our knowledge, it is the first time that the kesterite semiconductor has been investigated and employed as a photocatalyst in the photodegradation of the hazardous Congo red azo dye. Hence, emphasizing the novelty of the current work.

## 2. Materials and Methods

### 2.1. Hydrothermal Synthesis

The synthesis of CZTS nanoparticles was performed using a mixture of 2 mmol CuCl_2_ (Winkler, Langley, BC, Canada), 1 mmol ZnCl_2_ (Sigma-Aldrich, Oakville, ON, Canada), 1 mmol SnCl_2_ (Winkler P.A.), and 4 mmol CH_4_N_2_S (Merck, Rahway, NJ, USA) in a Teflon-coated stainless-steel reactor at 200 °C for 72 h. The pH value of the mother solution was measured employing a pH-meter (BANTE model PHS-25CW) with a value of 6.88 pH unities. The obtained nanoparticles were washed with 1:1 ethanol/water and centrifuged for 15 min at 4500 rpm (DLAB model DM0412). Finally, the CZTS nanoparticles were dried for 6 h at 60 °C in an oven in air atmosphere.

### 2.2. Characterization of CZTS Nanoparticles

The structural characterization of the CZTS phase was performed by X-ray diffraction (XRD) using a Bruker D8 ADVANCE diffractometer. The operating conditions were: CuKα radiation (30 mA, 40 kV, λ = 0.15418 nm), in Bragg–Brentano 45° mode, with a step of 0.01° and a step time of 34 s at room temperature.

Raman spectra were obtained using a Witec Alpha 300 confocal Raman microscope system equipped using an excitation laser wavelength of 785 nm and an electrically cooled CCD camera. The signal was calibrated using the 520 cm^−1^ line of a Si wafer and a 20× objective. The laser power on the samples was 2 mW. The resolution was set to 4 cm^−1^ and 10 scans with an integration time of 1 s were performed.

The chemical composition of the Cu_2_ZnSnS_4_ samples was studied via X-ray photoelectron spectroscopy (XPS) using a Physical Electronics (PHI) VersaProbe II spectrometer equipped with an Al Kα radiation source (1486.6 eV and 47.8 W).

Field emission scanning electron microscopy (FE-SEM) images of the CZTS samples were obtained on Helios Nanolab 650 Dual Beam equipment from FEI Company. For this, the powders were supported on conductive carbon tape inside the vacuum chamber. The analysis of the chemical composition of the formed structures was carried out using X-ray energy dispersion spectrometry (EDS). The equipment used was a QUANTAX 200 model from Bruker with XFLASH (EDS coupled to SEM equipment: Hitachi SEM SU-3500 of variable pressure with a detector 410-M). Samples for TEM were ultrasonically dispersed in 1 mL of ethanol. A small drop of the suspended solution was placed on a porous carbon film on a nickel screen and allowed to air dry. Transmission electron microscopy (TEM) and high-resolution transmission electron microscopy (HRTEM) images were obtained on a Talos F200X instrument. To obtain the mean sizes of the kesterite nanoparticles the ImageJ ver. 1.53t software has been used.

Optoelectronic properties of the nanoparticulate Cu_2_ZnSnS_4_ samples, i.e., optical and semiconducting properties have been verified through optical UV-visible absorption spectrometry measurements and by Mott–Schottky analysis, respectively, as is detailed below.

The optical properties were studied by UV-VIS molecular absorption spectra through the transmittance spectrum, using a SHIMADZU UV-2600 Spectrophotometer with a PC connection. The measurement range was from 400 nm to 900 nm at room temperature, with a scanning speed of 0.2 nm s^–1^, and 10 mg of CZTS nanoparticles suspended in ethanol were prepared.

Prior to the electrochemical analysis, thin films of CZTS were made using the drop-casting method, with a solution of the compound in ethanol to be placed in FTO (1.5 × 1 cm^2^). The films were dried for 4 h at 60 °C in an Ar atmosphere.

To electrochemically characterize the nanoparticles, cyclic voltammetry, and EIS were performed through the Mott–Schottky representation. The conventional cell with three electrodes was used: FTO/CZTS as the working electrode, Ag/AgCl (3M KCl) as the reference electrode, and a platinum wire as the auxiliary electrode, all immersed in a 0.1 M Na_2_SO_4_ solution as the supporting electrolyte. An AUTOLAB model PGSTAT 302 potentiostat/galvanostat and an FRA ZAHNER model ZENNIUM PP211 potentiostat/galvanostat were used for the measurements. The measurements were developed at room temperature. Mott–Schottky measurements were performed by applying an AC voltage with 10 mV amplitude with a frequency of 10 kHz at open circuit potential (0.03 V vs. Ag/AgCl 3 M), and analyzes were performed in a parallel circuit. In all cases, the area exposed to the supporting electrolyte was 1 cm^2^.

### 2.3. Photodegradation of Congo Red Azo Dye (CR)

For photodegradation, a 50 mL solution of 0.04 mM Congo red azo dye was prepared, to which 35 mg of CZTS nanoparticles were added. The solution was shaken in the dark for 30 min to generate adsorption–desorption equilibrium. Then, the solution was exposed to an ABET Technologies Model 11002 SunLite solar simulator (1000 W), and photodegradation was examined by monitoring the absorption spectra of Congo red azo dye solutions after various irradiation times using a Shimadzu UV-2600 UV-Vis spectrophotometer. The measurement wavelength range was 400–700 nm at room temperature, with a scanning speed of 0.2 nm s^−1^. Prior to photodegradation, a calibration curve of the CR dye was made, and the following equation was used to determine the *degradation efficiency*:

(1) $$Degradation\;efficiency\left(\%\right)=\frac{C_{0}-C_{t}}{C_{0}}\times 100$$

where *C*_0_ corresponds to the initial concentration of Congo red azo dye prior to exposure to illumination and *C_t_* corresponds to the concentration of Congo red dye after being exposed to solar illumination for a certain time *t*.

### 2.4. Photocatalyst Regeneration Study

The cyclability of the CZTS photocatalyst has been evaluated maintaining the experimental parameters described above. After each cycle, the nanocomposite was collected and washed with ethanol multiple times, dried for 30 min at 60 °C, and then reused for subsequent runs. Naturally, the loss of a portion of the photocatalyst has been observed, and then the amount of CZTS photocatalyst used in each cycle was not maintained.

## 3. Results

### 3.1. Structural, Morphological, and Surface Chemical Study

In order to determine structural and morphological properties, and also chemical surface composition, the prepared CZTS samples have been characterized by X-ray diffraction (XRD), Raman spectroscopy, X-ray photoelectron spectroscopy (XPS), field emission-scanning electron microscopy (FE-SEM) and high-resolution transmission electron microscopy (HR-TEM).

**Figure 1:**
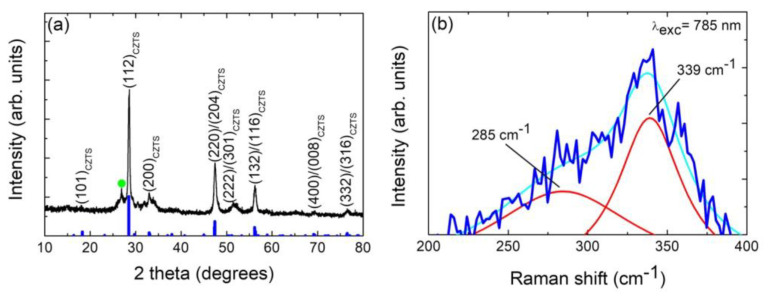
(**a**) X-ray diffraction pattern of a typical nanoparticulate Cu_2_ZnSnS_4_ sample. Diffraction planes are indicated for the Cu_2_ZnSnS_4_ phase (CZTS(hkl)). The signal marked with a green symbol (●) corresponds to the cassiterite SnO_2_ phase. The tetragonal kesterite JCPDS pattern (#04-023-6315) is also shown for comparison (Cu_2_ZnSnS_4_ JCPDS: thick blue bars). (**b**) Raman spectrum of nanoparticulate Cu_2_ZnSnS_4_ nanopowder sample measured with an excitation wavelength of 785 nm, indicating the characteristic peaks. The cyan line is the fit line while the red lines represent each component. The original spectrum is represented by a blue line.

Figure 1a shows a typical diffraction pattern of a hydrothermally grown nanoparticulate Cu_2_ZnSnS_4_ sample. For comparison, the XRD pattern of the standard tetragonal crystalline structure of Cu_2_ZnSnS_4_ (kesterite), JCPDS pattern #04-023-6315, is also provided. The XRD pattern exhibits four main intense diffraction peaks at 2 θ = 28.6°_,_ 2 θ = 32.9°, 2 θ = 47.6° and 2 θ = 56.3° ascribed to the (112), (200), (220)/(204) and (132)/(116) diffraction planes of the tetragonal crystalline structure of CTZS, respectively. In addition, four other faint diffraction peaks can be seen, which can also be ascribed to the kesterite CZTS phase. Results agree with other XRD structural studies of the kesterite phase previously reported in the literature [12,15,25,34]. Then, the XRD results indicate that the formed kesterite CZTS phase is well-defined, and the samples are polycrystalline. The presence of a very small impurity diffraction peak corresponding to SnO_2_ can be appreciated in the 2 θ range from 25° to 35° (see Figure 1a), indicating the presence of a minor SnO_2_ impurity secondary phase. Moreover, the broadening of the CZTS diffraction peaks demonstrates the nanocrystalline character of these samples. The average crystallite size was calculated from the full width at half maximum (FWHM) of XRD peaks by using the Scherrer formula [35,36]:

(2) $$D=\frac{k\lambda }{\beta cos\theta }$$

where *D* is the crystallite diameter, λ is the wavelength of the incident radiation, *k* = 0.89 is the shape factor, *θ* is the Bragg angle, and *β* is the full width at half maximum (FHWM) in radians. The average crystallite size evaluated from the (112) CZTS diffraction peak was ~18 nm, hinting at its nanocrystalline character.

Due to the similar structure of kesterite CZTS and some secondary phases like cubic ZnS and tetragonal Cu_2_SnS_3_, the CZTS main X-ray diffraction peak is easy to overlap with that of ZnS and Cu_2_SnS_3_ one [37,38,39,40]. Then, Raman scattering spectroscopy is generally adopted to find the co-existing secondary phases which cannot be identified effectively by X-ray diffraction [37,38,39,40]. To differentiate various phases, the first-order Raman spectra in the 200–400 cm^−1^ wavenumber region (see Figure 1b) has been analyzed by deconvolution using the Lorentzian peak fitting procedure. Figure 1b shows the presence of a main peak located at 339 cm^−1^ and a secondary one located at 285 cm^−1^ which corresponds to A_1_ and A vibrational modes of kesterite CZTS, respectively [38,40,41,42,43]. Thus, the results depicted in Figure 1b can allow us to highlight on one hand that the presence of these features are the characteristic peaks of CZTS and close to the reported values. On the other hand, no other peaks corresponding to ZnS (vibrational modes at 275 and 350 cm^−1^), cubic Cu_2_SnS_3_ (267, 303, and 356 cm^−1^), tetragonal Cu_2_SnS_3_ (297, 337, and 352 cm^−1^), Cu_2_S (267), SnS (189, 219, and 312 cm^−1^) and SnS_2_ (215 and 312 cm^−1^) are observed [37,40,44], which indicates that the synthesized CZTS presents a good quality with single phase.

**Figure 2:**
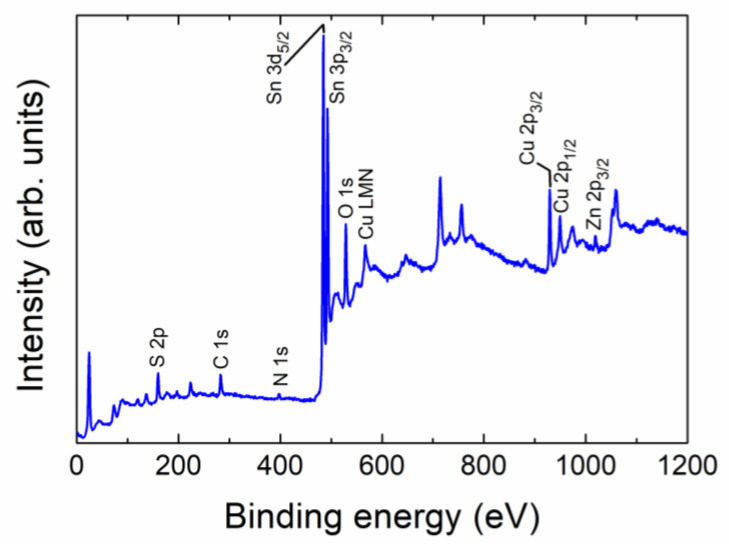
XPS survey spectrum for the synthesized particulate Cu_2_ZnSnS_4_ sample.

**Figure 3:**
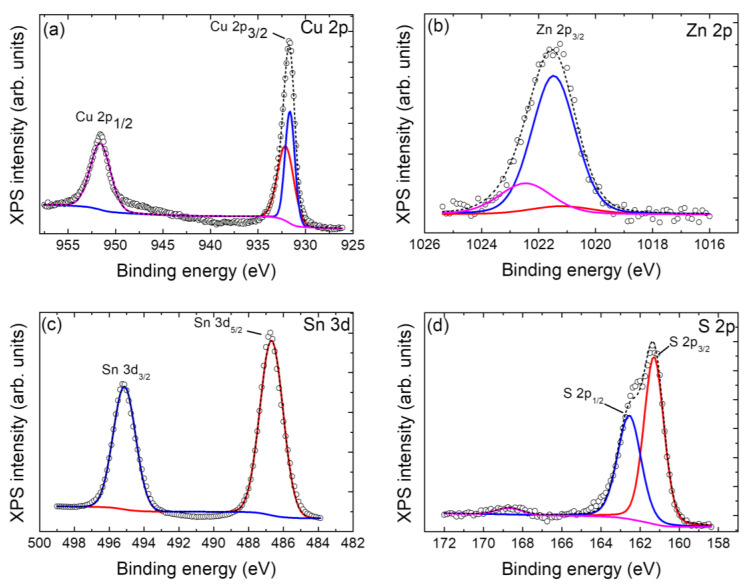
X-ray photoelectron spectroscopy high-resolution spectra for (**a**) Cu 2p, (**b**) Zn 2p, (**c**) Sn 3d, and (**d**) S 2p, for the Cu_2_ZnSnS_4_ sample. The short dashed black lines are the fit lines while the colored lines represent each component. The original spectra are represented as unfilled dots.

The synthesized nanoparticulate Cu_2_ZnSnS_4_ samples have been analyzed using XPS to check both the presence of Cu, Zn, Sn, and S and to verify the valence states of these constituent elements. The full XPS spectrum of the sample in Figure 2 shows the peaks of Cu, Zn, Sn, and S together with the C, N, and O peaks. It should be noted that the impurity peaks of C and O may be related to the reference or environmental contamination, and N to vestiges of the CH_4_N_2_S precursor. The valence state of elements in the CZTS was evaluated by high-resolution XPS spectra. Figure 3 shows the XPS high-resolution spectra. High-resolution spectra for all the core levels of interest (Cu 2p, Zn 2p^3/2^, Sn 3d^5/2^, and S 2p) were acquired, and also Cu LMM Auger transitions for better identification of the charge state for Cu, along with O 1s and C 1s. The spectra were calibrated at C 1s 284.8 eV. According to the Cu 2p spectra, a main peak is shown at 931.6 eV, and a secondary one at 932.1 eV, which can be related to Cu^+^ [45], probably due to the CuCl initial solution. As no satellites are visible at the spectra, no Cu^2+^ remains at the surface [4]. The assignment is also consistent with the calculated modified Auger parameter for each component reported to the Cu LMM Auger transition, taking values of 1849.3 and 1849.8 eV, respectively. According to the Zn 2p spectra, the main peak at 1021.5 eV corresponds to Zn^2+^. In reference to Sn 3d peaks, the mean peak appears at 486.7 eV, being related to SnO_2_ [45], possibly formed for the exposure of the samples to the air ambient oxygen. Finally, the S 2p peak was deconvoluted into two peaks: the main peak at 161.3 eV (S 2p_3/2_) is related to CuS [46], and the other at 162.5 eV (S 2p_1/2_) could be related to rests of the CH_4_N_2_S compound used for the prepared material. The valence states of Cu^+^, Zn^2+^, Sn^4+^, and S^2−^ confirm that the synthesized CZTS simple is well consistent with its stoichiometric formula of Cu_2_ZnSnS_4_ [14].

The morphological and microstructural features (i.e., detailed microstructure, size, and shape morphologies), and the chemical composition of the Cu_2_ZnSnS_4_ samples have been further investigated by field-emission scanning electron microscopy (FE-SEM) and transmission electron microscopy (TEM).

**Figure 4:**
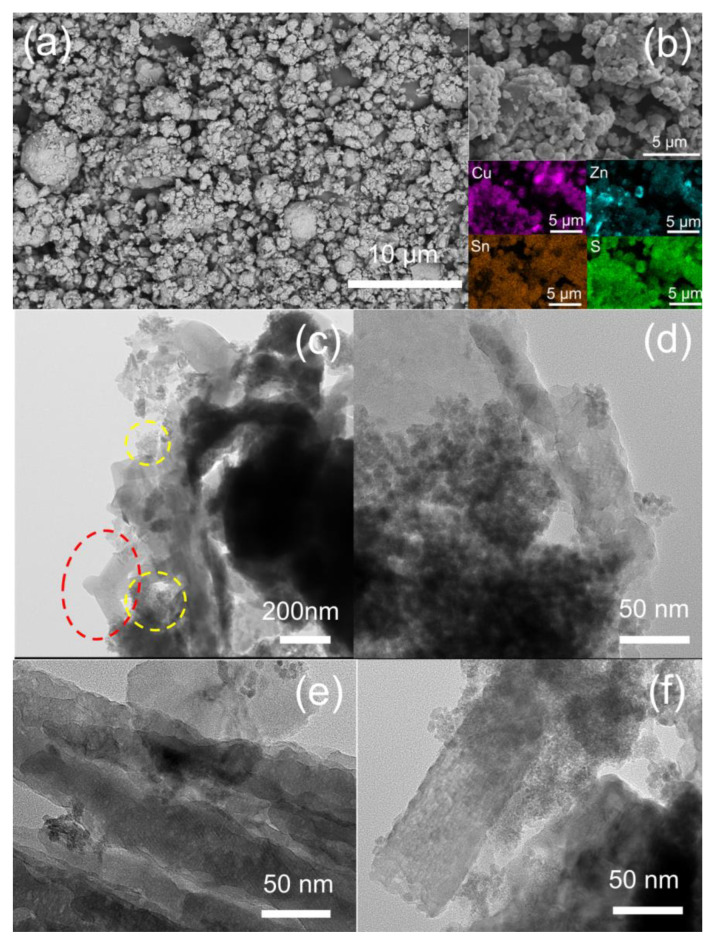
(**a**,**b**) FE-SEM plane-view micrograph images of a typical hydrothermally synthesized nanoparticulate Cu_2_ZnSnS_4_ sample at two different magnifications and different observed sample areas. EDX elemental mapping of Cu (violet), Zn (cyan), Sn (brown), and S (green) corresponding to Figure 4b FE-SEM micrograph image is depicted in four panels in the lower part of this (**b**). (**c**) Low-magnification TEM micrograph image of a typical hydrothermally synthesized nanoparticulate Cu_2_ZnSnS_4_ sample. Different CZTS nanocrystal shapes can be visualized: rod-like (indicated by a dashed red circle) and spherical forms (indicated by a dashed yellow circle). (**d**) High-magnification TEM micrograph of one of the zones highlighted by a dashed yellow circle in (**c**). (**e**,**f**) High-magnification TEM micrographs and different observed sample areas of the zone are highlighted by a dashed red circle in (**c**).

Figure 4a shows a low-magnification FE-SEM micrograph image of a typical hydrothermally synthesized Cu_2_ZnSnS_4_ sample showing the grain growth structure, revealing the loosely agglomerated CZTS nanoparticles with sizes comprised between 400 to 1200 nm (except for zones where bigger clusters of several microns can be appreciated). Figure 4b shows a higher magnification FE-SEM plan-view micrograph image of a Cu_2_ZnSnS_4_ sample. It can be seen that the prepared sample exhibits an aggregated morphology of clusters of particles, composed by the congregation of several smaller particles with sizes of about 140 to 200 nm. Moreover, to confirm the presence of all four elements in the nanopowder sample, the EDS elemental mapping has been conducted and it is shown in the four panels in the lower part of Figure 4a, revealing the presence of Cu, Zn, Sn, and S elements homogeneously distributed in the whole CZTS nanopowder sample. Figure 4c shows a TEM micrograph image of a typical nanoparticulate Cu_2_ZnSnS_4_ sample, where it can be appreciated the presence of CZTS nanocrystals in the form of spherical and rod-like shapes, as indicated. Similar CZTS nanoparticle shapes have been observed and reported in the literature for CZTS nanocrystals synthesized through a thermolysis route [47]. Figure 4d shows a higher magnification TEM micrograph of the zone highlighted by a dashed yellow circle in Figure 4c, depicting with more detail the presence of spherical CZTS nanocrystals, with an average size of 7 nm (size evaluation considering 100 nanoparticles that can be observed in the micrograph image). Figure 4e,f shows higher magnification TEM micrographs of the rod-like CZTS nanocrystals (see the zone highlighted by a dashed red circle in Figure 4c), exhibiting average diameter sizes in the range of 50 to 60 nm. According to XRD, the mean crystallite size of the CZTS sample is around 18 nm, giving then a mean value of the observed crystallite sizes obtained from the TEM micrographs. However, the obtained values from TEM images are more legitimate [18].

### 3.2. Optoelectronic Characterization

Optoelectronic properties of the nanoparticulate Cu_2_ZnSnS_4_ samples, i.e., optical and semiconducting properties have been verified through optical UV-visible absorption spectrometry measurements and by Mott–Schottky analysis, respectively.

**Figure 5:**
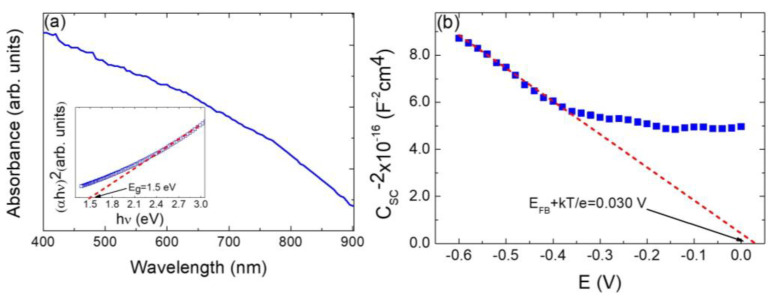
(**a**) UV-visible optical absorbance spectrum of a typical particulate Cu_2_ZnSnS_4_ sample. Inset: (*αhν*)^2^ vs. hν Tauc plot, for kesterite bandgap gap energy (*E_g_*) determination and corresponding linear fitting (red dashed line). (**b**) Mott–Schottky plot for a synthesized particulate Cu_2_ZnSnS_4_ sample. Red dashed line represents the fitted data in the linear range of the curve. The intercept of this line with the *x*-axis determinates the flat-band potential (*E_FB_*) as indicated. Measurements were carried out in 0.1 M Na_2_SO_4_ (pH = 6.5), with an AC frequency of 10 kHz.

Figure 5a shows the optical absorption spectrum of a typical nanoparticulate Cu_2_ZnSnS_4_ sample. From 400 to 700 nm, a moderate decreases of the optical absorption can be appreciated, whereas from 700 to 900 nm optical absorption decreases significantly. The optical bandgap (E_g_) and optical transition type of nanoparticulate Cu_2_ZnSnS_4_ samples have been determined from the Stern relation of near-edge absorption which is given as [36,48,49,50]:

(3) $$\alpha =\frac{A_{0}{\left(h\nu -E_{g}\right)}^{n}}{h\nu }$$

where *A*_0_ is a parameter related to the effective masses associated with the valence and conduction bands, and *hν* is the photon energy (where *ν* is the frequency, *h* is the Planck’s constant). m depends on the nature of band transitions, i.e., *n* = 1/2 or 2 for direct or indirect allowed transitions, respectively. The inset in Figure 5a shows the Tauc plot graph of (*αhν*)^2^ against hν so the optical bandgap can be determined. For all nanoparticulate Cu_2_ZnSnS_4_ samples, the optical absorption in the edge region can be well-fitted by this relation, demonstrating that the absorption edge is due to a direct allowed transition, which is in agreement with similar results that have been reported in the literature [12,51,52,53,54]. A direct energy optical bandgap of ca. 1.5 eV has been extracted by extrapolating the dashed line in the inset of Figure 5a to the energy axis. This value agrees very well with previous literature-reported values of 1.4 to 1.6 eV [12,26,51,52,53,54].

Mott–Schottky (M-S) analysis has been extensively incorporated by the field to assess the main operational parameters of semiconductor photoelectrodes, i.e., the flat-band potential, *E_FB_*, and the donor concentration, *N_D_* or acceptor concentration, *N_A_* (for an *n*-type semiconductor photoanode or a *p*-type photocathode) [55,56,57]. The Mott–Schottky equation simply relates the capacitance of the space-charge region (*C_SC_*) to the applied potential (*E*) relative to its *E_FB_* and other parameters of the semiconductor as follows [49,55,56,57,58]:

(4) $$\frac{1}{C_{SC}^{2}}=\frac{2}{\varepsilon \varepsilon_{0}eN_{D,A}}\left(E-E_{FB}-\frac{k_{B}T}{e}\right)$$

where ε and $\varepsilon_{0}$ are the dielectric constant and the vacuum permittivity, respectively, *e* is the electronic charge, *k_B_* is the Boltzmann constant, and *T* is the absolute temperature. Accordingly, a plot of *C_SC_*^−2^ vs. *E* should reveal *E_FB_* as the linearly extrapolated intercept with the abscissa, while *N_D,A_* will be proportional to the inverse of the slope. Figure 5b shows the plot of the inverse of the square of the serial capacitance as a function of the electrochemical potential for a nanoparticulate Cu_2_ZnSnS_4_ sample electrode. According to the Mott–Schottky equation (Equation (4)), a linear relationship with a negative slope is obtained, confirming that the nanoparticulate Cu_2_ZnSnS_4_ samples exhibit the semiconductor characteristic of a *p*-type material. A flat band potential value of 0.004 V (considering *k_B_T/e* = 0.026 V at 25 °C) and an acceptor carrier concentration *N_A_* = 1.5 × 10^14^ cm^−3^ have been obtained.

Thus, this Cu_2_ZnSnS_4_ *p*-type semiconductor has an extensive range of usages as a photocatalyst because according to the Shockley–Queisser limit, materials have the most visible light absorption of solar light irradiation in this bandgap region, so this bandgap makes it highly activated [59,60,61,62]. In fact, the photocatalysts having a bandgap value of around 1.5 eV shows the best photocatalytic efficiency [59,60,61,62].

### 3.3. Photocatalytic Activity Evaluation

**Figure 6:**
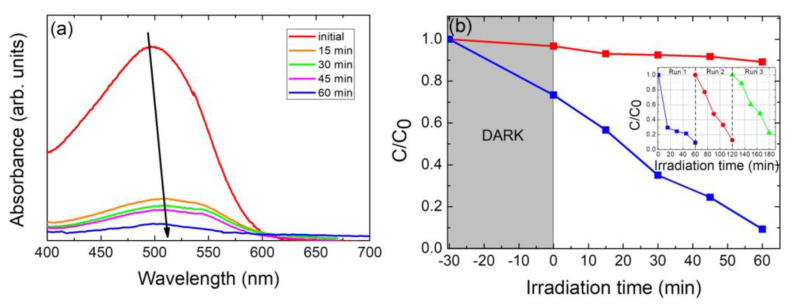
Photocatalytic degradation of Congo red azo dye in aqueous solution using nanoparticulate Cu_2_ZnSnS_4_ photocatalyst under AM1.5G simulated sunlight (100 mW cm^−2^) illumination. (**a**) Absorbance spectra as a function of illumination time, demonstrating the photodegradation of Congo red dye against CZTS (see direction black arrow). (**b**) Relative concentration (C/C_0_) versus time for photodegradation of Congo red azo dye in the presence (square blue symbols) and in the absence (square red symbols) of nanoparticulate Cu_2_ZnSnS_4_ photocatalyst, under AM1.5G, simulated sunlight (100 mW cm^−2^) illumination. Inset: recyclability of nanoparticulate Cu_2_ZnSnS_4_ sample as photocatalyst for the photodegradation of Congo red azo dye.

The photocatalytic performance of nanoparticulate Cu_2_ZnSnS_4_ samples has been evaluated by the photodegradation of Congo red azo dye in an aqueous solution under AM1.5G simulated sunlight (100 mW cm^−2^) illumination. Figure 6a shows the absorption spectra of this dye-aqueous solution, recorded in the wavelength range from 400 to 700 nm. As can be seen (see Figure 6a), the time-dependent absorbance spectra of photodegradation of Congo red dye aqueous solution show a characteristic peak at 497 nm, due to characteristic absorption to be assigned to the *n*-π* transition of the lone pair present on the N atom of the azo chromophore (–N=N–) [63]. The absorption peak of the dye diminished with illumination time (indicating that the concentration of Congo red dye decreases), indicating that a photodegradation takes place. Figure 6b shows the relative concentration (C/C_0_) changes of CR dye versus the solar simulator irradiation time. To validate the role of synthesized nanoparticulate Cu_2_ZnSnS_4_ photocatalyst, controlled experiments have also been carried out in the absence of photocatalyst; no significant degradation of CR has been observed (see Figure 6b, red line-symbols). Figure 6b shows that the photocatalytic activity of nanoparticulate Cu_2_ZnSnS_4_ after one-hour illumination, reaches a 90.2% degradation efficiency value. This value is significantly superior to that attained by TiO_2_ Degussa P25 which after one-hour illumination exhibits only a CR degradation efficiency of 70% [64,65,66]. This can be explained by taking into account that as CZTS is a narrow bandgap semiconductor (*E_g_* = 1.5 eV), under visible light illumination, it has the ability to degrade organic compounds in less time compared to TiO_2_ which presents a wide bandgap (*E_g_* = 3.2 eV) [12,18]. Moreover, it must be pointed out that crystallite size worth plays a significant role in improving the photocatalytic performance of photocatalysts. The surface area to volume proportion increases as the crystallite size value decreases, enhancing both the surface-active sites and the interfacial charge carrier transport rate. In the present case, the improved photocatalytic activity of the nanoparticulate Cu_2_ZnSnS_4_ photocatalyst could also then be attributed to its lowered crystallite size worth.

Furthermore, the observed degradation efficiency is higher than that obtained for the photodegradation of another azo dye such as Rhodamine B, where 83% or 51.7% could be obtained after 100 and 240 min, respectively [13,67]; the fact that may be related to the number of active sites for the generation of radical species given the nanocrystalline nature of our Cu_2_ZnSnS_4_ phase.

The CR photodegradation has been fitted to a pseudo-first-order kinetic model that can be expressed as follows:

(5) $$ln\frac{C_{0}}{C}=kt$$

where *k* is the kinetic rate constant, *C*_0_ is the initial concentration, and *C* is the concentration at given time *t*. A constant *k* of 0.01 min^−1^ was obtained, being comparable to that exhibited by Degussa P25 TiO_2_ [68,69,70].

The optimal photocatalyst portrays the best performance, and its photocatalytic property remains after several photodegradation cycles; in fact, the stable recyclability of one photocatalyst is vital for future practical applications [18,62]. Then, recycling photodegradation experiments of nanoparticulate Cu_2_ZnSnS_4_ photocatalyst have been carried out, as shown in the inset of Figure 6b. The measurement was repeated three times to evaluate the stability and reusability of the photocatalyst (see the Experimental section for details). The inset of Figure 6b gives the degradation rate for the 3 cycles. The photocatalytic performance remained stable after the third cycle (a minor deviation (~14%) from the initial degradation efficiency has been observed), which further confirms that the stability and reusability of nanoparticulate Cu_2_ZnSnS_4_ photocatalyst are reasonable. However, this decrease in the dye photo-degradation efficiency after several runs can be mainly due to the CZTS photocatalyst mass loss during the successive filtering and washing processes in between each photodegradation run cycle (processes carried out in order to remove and reuse the photocatalyst material).

## 4. Conclusions

The hydrothermal method proved to be a simple, low-cost, low-pollution route through which it was possible to obtain CZTS nanoparticles. XRD, RAMAN, XPS, and EDS confirmed that the structure and composition of the synthesized nanoparticles correspond to those of pure Cu_2_ZnSnS_4_. The formation of spherical and rod-like nanocrystals with mean sizes of 7 nm and 50–60 nm, respectively, has been found by TEM analysis. The EDS results confirmed that the synthesized nanopowder exhibited a homogeneous elemental distribution in the whole CZTS sample. From UV-visible data, the bandgap of CZTS films has been determined to be 1.5 eV, which is optimal for photocatalytic applications. The CZTS phase proved to be an excellent photo-catalyst for CR where 90.2% degradation could be achieved in just 60 min under solar simulation light irradiation, better than other photo-catalysts such as TiO_2_, widely recognized for its good efficiency. The reusability experiment confirms the stability of the photocatalytic activity of the as-prepared CZTS nanopowder after three runs of the photodegradation process.

## Data Availability

Not applicable.
